# Does lithium reduce acute suicidal ideation and behavior? A protocol for a randomized, placebo-controlled multicenter trial of lithium plus Treatment As Usual (TAU) in patients with suicidal major depressive episode

**DOI:** 10.1186/s12888-015-0499-5

**Published:** 2015-05-19

**Authors:** U. Lewitzka, B. Jabs, M. Fülle, V. Holthoff, G. Juckel, I. Uhl, S. Kittel-Schneider, A. Reif, C. Reif-Leonhard, O. Gruber, B. Djawid, S. Goodday, R. Haussmann, A. Pfennig, P. Ritter, J. Conell, E. Severus, M. Bauer

**Affiliations:** Department of Psychiatry and Psychotherapy, University Hospital Carl Gustav Carus, Technische Universität Dresden, Fetscherstr. 74, Dresden, D-01307 Germany; Psychiatric Department of the Municipal Hospital Dresden-Neustadt, Dresden, Germany; Department of Psychiatry, Psychotherapy and Psychosomatic Medicine, Alexianer Krankenhaus Hedwigshöhe, Berlin, Germany; Department of Psychiatry, Psychotherapy, Prevention Medicine, LWL-University Clinic Bochum, Ruhr University Bochum, Bochum, Germany; Department of Psychiatry, Psychosomatic Medicine and Psychotherapy, Goethe-Universit, Frankfurt, Germany; Center for Translational Research in Systems Neuroscience and Psychiatry, Clinic for Psychiatry and Psychotherapy, University Medical Center Göttingen, Göttingen, Germany; Coordination Centre for Clinical, Trials (KKS) Dresden, Medical Faculty Carl Gustav Carus, TU Dresden, Dresden , Germany; Department of Epidemiology Dalla Lana School of Public Health, University of Toronto, Toronto, Canada

**Keywords:** Randomized controlled trial, Lithium, Suicidal thoughts, Suicidal behavior, Affective disorders

## Abstract

**Background:**

Lithium has proven suicide preventing effects in the long-term treatment of patients with affective disorders. Clinical evidence from case reports indicate that this effect may occur early on at the beginning of lithium treatment. The impact of lithium treatment on acute suicidal thoughts and/or behavior has not been systematically studied in a controlled trial. The primary objective of this confirmatory study is to determine the association between lithium therapy and acute suicidal ideation and/or suicidal behavior in inpatients with a major depressive episode (MDE, unipolar and bipolar disorder according to DSM IV criteria). The specific aim is to test the hypothesis that lithium plus treatment as usual (TAU), compared to placebo plus TAU, results in a significantly greater decrease in suicidal ideation and/or behavior over 5 weeks in inpatients with MDE.

**Methods/Design:**

We initiated a randomized, placebo-controlled multicenter trial. Patients with the diagnosis of a moderate to severe depressive episode and suicidal thoughts and/or suicidal behavior measured with the Sheehan-Suicidality-Tracking Scale (S-STS) will be randomly allocated to add lithium or placebo to their treatment as usual. Change in the clinician administered S-STS from the initial to the final visit will be the primary outcome.

**Discussion:**

There is an urgent need to identify treatments that will acutely decrease suicidal ideation and/or suicidal behavior. The results of this study will demonstrate whether lithium reduces suicidal ideation and behavior within the first 5 weeks of treatment.

**Trial registration:**

ClinicalTrials.gov identifier: NCT02039479

## Background

Over the past several decades our knowledge about suicidal behavior has greatly increased. Research could prove the importance of the interplay between neurobiological, social, psychological, cultural and environmental factors for the development of suicidal behavior. Epidemiological studies have led to more information about risk and protective factors in general population and in patients with psychiatric diseases. As it is indicated in the first report on suicide prevention (WHO 2014 [1]) many universal, selected and indicated strategies have been implemented worldwide to improve the knowledge about suicide, increase the access to health care professionals, train the gatekeepers and therefore lead to an improvement in identification and management of people at risk. Twenty eight countries have established national suicide prevention programs dedicated to suicide research and prevention; however, there is still a need to expand suicide prevention research and anti-suicidal strategies. Lithium treatment has been a popular research avenue given its often replicated anti-suicidal effects and different effects on the human body.

For example, studies have investigated the influence of lithium therapy on neuroprotection [2], inflammation [3], genetic changes [4] and its anti-suicidal properties. Lithium is approved in International Guidelines for the maintenance treatment of bipolar disorder [5–7] and has shown to be effective in prevention of depressive and manic episodes. Long-term treatment with lithium has been shown to be effective in preventing both suicide and attempted suicide in patients with unipolar and bipolar depression. Further, lithium appears to be more effective in reducing suicide compared to other pharmacological treatments effective in preventing mood episodes (e.g. valproate) as well as compared to placebo. One piece of evidence derives from randomized controlled trials investigating the mood-stabilizing effect of lithium. More specific information, however, has been gained from the careful analysis of cohorts of well documented patients having received lithium over many years under constant systematic monitoring (for a comprehensive overview please see Lewitzka et al., submitted [8]). As patients with mood disorders have a 30-fold greater risk of suicide compared to the general population, it is of great importance to systematically study the acute effects of lithium in a controlled setting.

So far little is known about the mechanism behind this effect. Neurobiological research has focused on lithium’s influence on neurotransmitters such as serotonin [9] or noradrenalin and dopamine [10]. Lithium’s influences on the cortisol stress hormone system [11], the γ-aminobutyric-acid [12], second messenger systems such as the inositol metabolism and glycogen synthase kinase 3 have also been investigated [13]. Lithium has demonstrated to have an effect on impulsivity and aggression via several pathways within the nerve cell [14, 15] which is discussed as one of the underlying mechanisms behind the anti-suicidal efficacy.

As suicide research has to deal with specific methodological difficulties the design and development of study protocols needs to be cautious. Ethical and statistical issues need thoughtful consideration.

In 2005, the worldwide first placebo-controlled randomized trial designed specifically to investigate the influence of lithium on suicidal behavior was conducted and published in 2008 by Lauterbach et al. [16]. Survival analysis showed no significant difference in suicidal acts between lithium and placebo-treated individuals (adjusted hazard ratio 0.517; 95 % CI 0.18–1.43); however, post hoc analysis revealed that all completed suicides had occurred in the placebo group accounting for a significant difference in incidence rates (*P* = 0.049). Within a treatment period of one year, no suicides occurred within the lithium group (*n* = 84) compared to three suicides within the placebo group (*n* = 83). No significant differences were found in terms of suicide attempts (seven suicide attempts each in the placebo and lithium group). To date, no study has investigated how fast this anti-suicidal effect appears. It is unclear where in the course of treatment this effect arises.

The primary aim of the present study is to determine whether lithium exerts an anti-suicidal effect through a decrease in suicidal thoughts and/or behavior within the first 5 weeks of treatment. The secondary aim of the study is to determine whether lithium influences suicidal thoughts and/or behavior through the indirect path of improving depressive symptoms, anxiety or impulsivity.

### Methods/Design

The present study is a multi-center, 4 year, randomized placebo-controlled double-blind trial. Patients with unipolar or bipolar disorder suffering from a major depressive episode with suicidal thoughts and/or behavior according to DSM IV criteria will be randomly allocated to lithium therapy plus treatment as usual versus placebo treatment plus treatment as usual. The hypothesis to be tested is that lithium treatment plus treatment as usual leads to a significant reduction of suicidal thoughts and/or behavior compared to placebo therapy plus treatment as usual within 5 weeks of treatment. The primary end point of this study is the change in Sheehan Suicidality Tracking Scale (S-STS, [17]) between the beginning of treatment and after a treatment episode of 5 weeks. Further endpoint in this study are changes in the Columbia-Suicide Severity Rating Scale (C-SSRS, [18]), the Montgomery Asberg Depression Rating Scale (MADRS, [19]), the Hamilton Anxiety Scale (HAM-A, [20]), the Clinical Global Impression Scale (CGI, [21]) and the Barratt Impulsivity Scale (BIS, [22]) from initial treatment to 5 weeks post treatment. A comparison of total scores in the C-SSRS, MADRS, HAM-A and the Young Mania Rating Scale (YMRS, [23]) within the participating centers will be conducted. In addition, the number and dosage of concurrent medication and number of psychotherapy hours will be explored as secondary endpoint. Weekly changes in the C-SSRS, MADRS, HAM-A and YMRS within the observation period and percentage of days where the S-STS Scale score equals zero will be reported. Two-hundred and fifty-four patients will be included with, thereof 127 in each treatment group. Recruitment started on October first, 2013 and will end on September 30^th^, 2017. Treatment includes 5 weeks of treatment plus 1 week of follow-up. For a detailed description of the inclusion and exclusion criteria, please see Table 1.

**Table 1:** In-and exclusion criteria

Inclusion criteria	Exclusion criteria
- Male and female patients at least 18 years of age and older - Presenting with a depressive episode (MDE, uni/bipolar), moderate to severe according to DSM IV criteria - Admitted as an inpatient - Suicidal thoughts and/or behavior defined by a score ≥ 8 in the S-STS scale and a score ≥ 20 in the MADRS Scale at time of screening and baseline - Written informed consent	- Pre-treatment with lithium within the last six months before inclusion in the study - Patients after compulsory hospitalization or patients unable to understand the study protocol - Diagnosis of antisocial and/or Borderline personality disorder, alcohol and drug dependency (positive drug screening) - Contraindication following drug approval such as severe heart failure (arrhythmias), severe renal dysfunction, Addison’s disease and others - History of hypersensitivity towards components of trial medication - Pregnant or lactating women, women attempting to get pregnant, and women not practicing a reliable method of contraception

### Study population

**Fig. 1:**
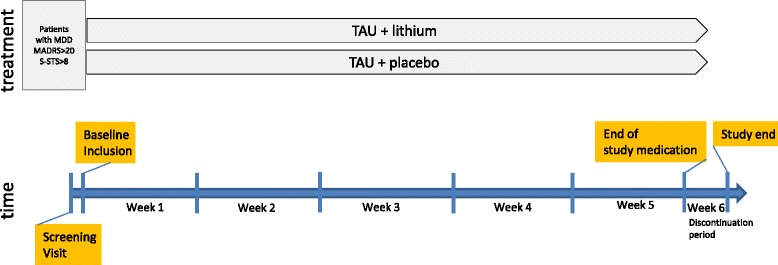
Trial flow

Patients will be recruited from six psychiatric hospitals (Dresden University Clinic, Dresden Neustadt, Berlin, Bochum, Frankfurt, Göttingen) in Germany. Specifically, patients will be recruited upon admission. All patients meeting inclusion criteria will be informed about the study by one of the investigators. Following informed written consent, randomization will be conducted by a central block design, allocating the participant to one of the two treatment groups. Figure 1 shows the trial time-line.

### Intervention/Course

Patients allocated to the lithium treatment group will receive an oral starting dose of 295 mg lithium carbonate on the first day, increased to 885 mg on the second day, increasing the dosage to serum concentrations between 0,6 and 1,0 mmol/l regularly given twice daily for a total of 5 weeks. Lithium levels will be measured 3 days after first intake and every 3 days after dose changes. Those being allocated to the placebo group will receive the placebo with a virtual concentration level between 0,6 and 1,0 mmol/l also for 5 weeks. The psychiatrist who dispenses and recommends dose adjustments of both lithium and placebo will be different from the patient’s treating psychiatrist and will be blind to the patients study group. Both groups will receive treatment as usual for their depressive symptoms. The average hospital stay for patients with depression and suicidal thoughts and/or behavior in Germany is about 4 weeks. If the patient is discharged from the hospital before the end of the study, the patient will continue in the study as an outpatient using the same procedure.

In total there will be nine study visits. The screening visit (V1, day −3 to 0) will involve confirmation of eligibility, informed written consent, anamnesis, an ECG, a physical exam, and a blood sample for lithium level measurement. Afterwards (up to 3 days later) a baseline screening visit (V2) at day 0 will occur. At the baseline visit, eligibility will be re-confirmed in addition to randomization and beginning of treatment in the evening. Three days after the baseline visit (V2), weekly visits (V3–V7) will occur involving psychometric testing, examination of vital signs and concurrent medication. Any adverse events as well as lithium levels will be carefully documented at each visit. The end of treatment will be on the day of the last weekly visit (V8). Within 1 week following visit 8 will be the final follow-up visit. This visit will involve safety measurements such as vital signs, ECG and a blood sample.

### Assessments

#### Clinician administered scales

The Mini International Neuropsychiatric Interview (MINI, [24]) as well as the structured clinical interview for DSM-IV axis II personality disorders (SKID II, [25]) will be used for diagnosis. To assess suicidal thoughts and/or behavior, the Sheehan Suicidality Tracking Scale (S-STS) is used, both as clinician and self-reported. The S-STS scale was selected because it maps directly to the Columbia Classification Algorithm of Suicide Assessment (C-CASA) coding system, endorsed by the FDA. The S-STS evolved from the Suicidality Module of the MINI Structured Diagnostic Interview for DSM-IV and is a prospective self or clinician report scale. The eight item scale assesses treatment-emergent suicidal ideations and treatment-emergent suicidal behaviors. Each item of the S-STS is scored on a 5-point Likert scale (0 = not at all, 1 = a little, 2 = moderately, 3 = very, and 4 = extremely) and it takes approximately 1 to 2 min to complete. Data from the Sheehan-STS can be analyzed as individual item scores, suicidal ideation subscale score, suicidal behavior subscale score, and total score.

As suicidal thoughts and/or behavior are primary outcomes in this study we also included the Columbia-Suicide Severity Rating Scale (C-SSRS) which has demonstrated good convergent and divergent validity with other multi-informant suicidal ideation and behavior scales and demonstrates high sensitivity and specificity for suicidal behavior classifications. The C-SSRS distinguishes between the domains of suicidal ideation and suicidal behavior. Four constructs are measured. The first is the severity of ideation, which is rated on a 5-point ordinal scale in which 1 = wish to be dead, 2 = nonspecific active suicidal thoughts, 3 = suicidal thoughts with methods, 4 = suicidal intent, and 5 = suicidal intent with plan. The second is the intensity of ideation subscale, which comprises 5 items, each rated on a 5-point ordinal scale: frequency, duration, controllability, deterrents, and reason for ideation. The third is the behavior subscale, which is rated on a nominal scale that includes actual, aborted, and interrupted attempts; preparatory behavior; and non-suicidal self-injurious behavior. The fourth subscale describes lethality, containing the assessment of actual attempts and actual lethality. It is rated on a 6-point ordinal scale, and if actual lethality is zero, potential lethality of attempts is rated on a 3-point ordinal scale. The C-SSRS is frequently recommended by various international agencies such as the FDA or WHO.

The Montgomery Asberg Depression Rating Scale (MADRS) and the Hamilton Anxiety Rating Scale (HAM-A) assess depressive and anxiety symptoms. Symptom severity, treatment response and the efficacy of treatment are measured using the Clinical Global impression scale (CGI).

#### Patient administered scales

To investigate whether the psychological construct of impulsivity is associated with suicidality, the Barratt Impulsiveness Scale (BIS) will be applied at weekly intervals during the study. The BIS is a widely used measure of impulsiveness and includes 30 items that are scored to yield six first-order factors (attention, motor, self-control, cognitive complexity, perseverance and cognitive instability impulsiveness) and three second-order factors (attentional, motor, and non-planning impulsiveness). The patients will be also asked to fill out the S-STS as a self-rating instrument on a daily basis.

### Power analysis and sample size calculation

A comparable study published by Khan et al. [26] of a 6-week double-blind trial in 40 patients found a score reduction of 4.8 (SD = 5.1) in the clinician administered S-STS in the placebo group, while a score reduction of 6.7 (SD = 5.8) was found among the lithium group The assumption that all lithium patients achieve a therapeutic serum-level in the current study seems to be reasonable due to the considerable higher lithium dose in comparison with the Khan et al. study. Therefore, these values have been used as estimates for the expected group difference in this study.

With an alpha level of 0.05, and statistical power of 0.8, sample size estimation for a two-sided *t*-test with unequal variances results in a required number of 131 patients per group. Nevertheless, a *t*-test has considerably less power than an analysis of covariance (ANCOVA), which will be used in this analysis. Borm et al. [27] showed for sample size n and a baseline-follow-up correlation rho, that an ANCOVA with (1-rho^2)*n subjects has the same power as the corresponding *t*-test. There is no published data on test-retest correlations of the S-STS to our knowledge; however, a recalculation of data from the “Basis-Studie Kompetenznetz Depression” [28] with 1014 naturalistically treated depressed patients showed correlations from 0.36 to 0.54 for the self-report S-STS between baseline and week 8. Therefore, a conservative assumption of 0.3 for the expected correlation was chosen, which yields a number of 120 patients per group. These patients will form the modified Intention to Treat (ITT) sample including all patients who will be randomized and not discontinue from the study before any efficacy measurement. A rate of approximately 5 % of randomized patients dropping out for any reason before entering the ITT sample is presumed, which results in 127 patients needed to be randomized per group. Therefore, 508 patients will be required to be screened assuming a rate of 50 % of screened patients fulfilling the inclusion criteria. We are expecting good adherence to the intervention as this will not result in major side effects. However, we anticipate that there will be about 5 % loss to follow-up, and this is accounted for in the sample size estimation.

As the study is a multi-center trial, randomization will be carried out by a centralized computer randomization directly after screening and obtainment of written informed consent at visit 2 (baseline). Study participants and the treating mental health team on the inpatient unit will be blinded for group assignment. A rater-training (including assessment of inter-rater reliability) will be provided before the study commences to minimize differences between centers.

### Statistical analysis

Descriptive statistics using proportions, means and standard deviations will be reported where appropriate. All efficacy outcome measures will be analyzed on an intent-to-treat (last observation carried forward) basis and study completer basis. Results will be presented with 95 % confidence intervals where applicable. Proportions of adverse events will be summarized. All statistical tests will be two-sided using an alpha level of 0.05. Analyses will be adjusted for age and gender when there is a significant difference between the groups at baseline.

#### Primary outcome measures

An analysis of covariance (ANCOVA) model will be used to compare the final clinician administered S-STS scores between the groups, including the initial score as a covariate.

#### Secondary outcome measures

ANCOVA models will be also used for continuous variables, including the initial S-STS score as a covariate. The number of days with a patient-rated S-STS score of 0 will be calculated for each patient, and compared between the groups. Because this outcome measure is a count variable, Poisson regression will be applied. Cumulative regression models will be applied for the comparison between groups for the BIS, CGI and the C-SSRS-items concerning severity and intensity of ideation. For the analysis of longitudinal data, (i.e. the modelling of weekly score change), mixed regression models will be used.

### Data and safety management

Pre-trial visits will be performed at all sites by the coordination Centre of Clinical Trials (KKS) Dresden to evaluate the qualification of the sites, and the recruitment of the necessary number of patients. To ensure the study is conducted in accordance with the protocol and Good Clinical Practice (GCP), monitoring will be performed by experienced personnel from the KKS Dresden at each site in appointed time intervals (per site, once at initiation of the study, once after the first subject was randomized, once when the last subject completed the study), for a total of 11 regular monitoring visits per site. The monitoring process will be performed according to the SOPs of the sponsor of this trial, the Dresden University of Technology. The monitor will check completeness and plausibility of the data and will align the study data to the original data (Source Data Verification). This will be accomplished by accessing the subject’s charts. As part of the consent, subjects give permission for study personnel to access their medical charts. The monitor will ensure the basic claim of integrity and protection of the subject’s privacy. All trials with the Dresden University of Technology will be audited. All assessed data will be recorded in the eCRFs with an audit trail by authorized personnel. A list of signs and identification codes of all personnel that will enter data into the CRFs will be stored in the Trial Master File. Corrections are only allowed to be processed by these persons. All study data will be stored for 10 years and will be destroyed afterwards.

This trial will be supervised by a scientific advisory board including members experienced in assuring data quality and safety (DSMB). These members have supervised the development of this protocol and will supervise the progress of the trial, including decision making on whether to perform interim analyses or modify/stop the trial in case of unforeseeable problems. The members of this board are completely independent of the coordinating investigators and the medical institution involved. As per protocol, all serious adverse events and reactions will be documented by the investigators, and due to German Drug law, evaluated and sent to the sponsor, or if necessary to the regulatory authority.

### Ethical considerations

An extensive number of patients with depression have suicidal thoughts and/or behavior during their acute episodes of illness. All patients will receive TAU. Because of the long history of lithium in the treatment of mood disorders with well-known (side) effects, the risks associated with lithium treatment in the proposed study protocol are considered low, especially as this is only a 5-week trial. The potential benefits of this trial clearly outweigh the potential risks associated with lithium treatment. Finally, it has been shown that patients at high risk for suicide can safely be included in clinical trials such as this one if proper precautions are followed. In addition, these patients need to be included to enable premarket assessments of the risks and benefits of medications related to suicidal ideation, suicidal behavior, and suicide in this population [29].

The study has been approved by the Ethics Committee in Dresden (coordinating centre) on August, 23th 2013 (EUDRACT number 2013-000970–31) and was also approved for all participating centres (Dresden University Clinic, Dresden Neustadt, Berlin, Bochum, Frankfurt, Göttingen).

## Discussion

Lithium has shown to have suicide-preventing effects in patients with affective disorders over the long-term course. No study has investigated whether this effect occurs at the beginning of treatment or develops after months of ongoing lithium therapy. The results of this study should indicate whether lithium leads to a decrease in suicidal thoughts and/or behavior within the beginning of the treatment (5 weeks).

There is a critical need to identify treatments that will acutely decrease suicidal ideation and/or behavior. Lithium therapy is widely available and inexpensive. The potential benefits of lithium therapy on acute suicidal ideation and/or behavior in patients with affective disorders need to be clarified.

In designing this study, we aimed to be as close as possible to a real-world clinical setting. The recruiting centers are a mix of university and community based psychiatric hospitals which represents a broad spectrum of patients with affective disorders. We are aware that the TAU condition may cause additional heterogeneity, as this can vary from center to center, or even within one center through different approaches of the treating psychiatrists.

This proposed study has several limitations: i) suicidal patients are generally excluded from clinical (especially pharmacological) trials. Therefore special attention and care is needed to minimize the risk in this high-risk population while undergoing a clinical trial. ii) Patients with acute suicidal crisis admitted to the hospital and treated with combined pharmacological and psychological treatments sometimes show a sudden improvement in terms of their suicidality, especially in those who were intoxicated at the time of admission. In the literature this effect is well-described as a suicide-cathartic effect after a suicide attempt [30]). Suicidality fluctuates in patients with depressive symptoms from the day of admission to a few days later when they usually are being informed about the study. As Davis et al. [31] stated, interpersonal support (attention from loved ones, and benefits from therapy) may be a mechanism by which a patient experiences immediate relief, and therefore decreased suicidal ideation/behavior. iii) Currently, no approved medication exists to treat suicidality. Patients may express concerns that they will be receiving a medication that is not approved for their specific symptoms and may refuse to participate.

Despite these challenges we anticipate that the results of this study (coming from a real world setting), will provide novel information that could lead to better pharmacological treatment and prevention strategies for patients with an acute major depressive episode with suicidal ideation and/or behavior, and may help reduce the immense individual distress for families in which a member is suffering from depression with suicidality.

## References

[1] WHO. Preventing suicide: a global imperative. [http://www.who.int/mental_health/suicide-prevention/world_report_2014/en/].

[2] Machado-Vieira R, Manji HK, Zarate CA (2009). The role of lithium in the treatment of bipolar disorder: convergent evidence for neurotrophic effects as a unifying hypothesis. Bipolar Disorder.

[3] Quiroz JA, Machado-Vieira R, Zarate CA, Manji HK (2010). Novel insights into lithium’s mechanism of action: neurotrophic and neuroprotective effects. Neuropsychobiology.

[4] McCarthy MJ, Leckband SG, Kelsoe JR (2010). Pharmacogenetics of lithium in bipolar disorder. Pharmacogenetics.

[5] Pfennig A, Bauer M (2013). S3guidelines on bipolar disorders are contemporary and important instruments for clinical practice. Nervenarzt.

[6] Grunze H, Vieta E, Goodwin GM, Bowden C, Licht RW, Möller HJ (2013). The World Federation of Societies of Biological Psychiatry (WFSBP) Guidelines for the Biological Treatment of Bipolar Disorders: Update 2012 on the long-term treatment of bipolar disorder. World J Biol Psychiatry.

[7] Lam RW, Kennedy SH, Grigoriadis S, McIntyre RS, Milev R, Ramasubbu R (2009). Canadian Network for Mood and Anxiety Treatments (CANMAT) clinical guidelines for the management of major depressive disorder in adults III: Pharmacopsychiatry. J Affect Disord.

[8] Lewitzka U, Severus E, Bauer R, Ritter P, Müller-Oerlinghausen B, Bauer M. The suicide prevention effect of lithium: more than 20 years of evidence. Submitted Int J Bip Disord 10.1186/s40345-015-0032-2 PMC4504869 26183461

[9] Price LH, Charney DS, Delgado PL, Heninger GR (1989). Lithium treatment and serotonergic function. Neuroendocrine and behavioural response to intravenous tryptophan in affective disorder. Arch Gen Psychiatry.

[10] Ernst CL, Goldberg JF (2004). Antisuicide properties of psychotropic drugs: a critical review. Harv Rev Psychiatry.

[11] Bschor T, Ritter D, Winkelmann P, Erbe S, Uhr M, Ising M (2011). Lithium monotherapy increases ACTH and cortisol response in the DEX/CRH test in unipolar depressed subjects. A study with 30 treatment-naïve patients. PLoS One.

[12] Motohashi N (1992). GABA receptor alterations after chronic lithium administration. Comparison with carbamazepine and sodium valproate. Prog Neuropsychopharmacol Biol Psychiatry.

[13] Kovacsics CE, Gottesman II, Gould TD (2009). Lithium’s antisuicidal efficacy: elucidation of neurobiological targets using endophenotype strategies. Annu Rev Pharmacol Toxicol.

[14] Müller-Oerlinghausen B, Lewitzka U (2010). Lithium reduces pathological aggression and suicidality: a mini-review. Neuropsychobiology.

[15] Mühlbauer HD, Müller-Oerlinghausen B (1985). Fenfluramine stimulation of serum cortisol in patients with major affective disorders and healthy controls: further evidence for a central serotonergic action of lithium in man. J Neural Transm.

[16] Lauterbach E, Felber W, Müller-Oerlinghausen B, Ahrens B, Bronisch T, Meyer T (2008). Adjunctive lithium treatment in the prevention of suicidal behaviour in depressive disorders: a randomised, placebo-controlled, 1-year trial. Acta Psychiatr Scand.

[17] Coric V, Stock EG, Pultz J, Marcus R, Sheehan DV (2009). Sheehan Suicidality Tracking Scale (Sheehan-STS): preliminary results from a Multicenter Clinical Trial in Generalized Anxiety Disorder. Psychiatry (Edgmont).

[18] Posner K, Brown GK, Stanley B, Brent DA, Yershova KV, Oquendo MA (2011). The Columbia-Suicide Severity Rating Scale: initial validity and internal consistency findings from three multisite studies with adolescents and adults. Am J Psychiatry.

[19] Montgomery SA, Asberg M (1979). A new depression scale designed to be sensitive to change. Br J Psychiatry.

[20] Hamilton M (1959). The assessment of anxiety states by rating. Br J Med Psychol.

[21] Guy W, National Institute of Mental Health (1976). Clinical Global Impressions. ECDEU assessment.

[22] Barratt ES (1965). Factor analysis of some psychometric measures of impulsiveness and anxiety. Psychol Rep.

[23] Young RC, Biggs JT, Ziegler VE, Meyer DA (1978). A rating scale for mania: reliability, validity, sensitivity. Br J Psychiatry.

[24] Sheehan DV, Lecrubier Y, Sheehan KH, Amorim P, Janavs J, Weiller E (1998). The Mini-International Neuropsychiatric Interview (M.I.N.I.): The development and validation of a structured diagnostic psychiatric interview for DSM-IV and ICD-10. J Clin Psychiatry.

[25] Spitzer RL, Williams JBW (1987). Structured clinical interview for DSM-III-R personality disorders.

[26] Khan A, Khan SR, Hobus J, Faucett J, Mehra V, Giller EL (2011). Differential pattern of response in mood symptoms and suicide risk measures in severly ill depressed patients assigned to citalopram with placebo or citalopram combined with lithium: role of lithium levels. J Psychiatr Res.

[27] Borm GF, Fransen J, Lemmens WA (2007). A simple sample size formula for analysis of covariance in randomized clinical trials. J Clin Epidemiol.

[28] Seemüller F, Riedel M, Obermeier M, Bauer M, Adli M, Kronmüller K (2010). Outcomes of 1014 naturalistically treated inpatients wiht major depressive episode. Eur Neuropsychopharmacol.

[29] Meyer RE, Salzman C, Youngstrom EA, Clayton PJ, Goodwin FK, Mann JJ (2010). Suicidality and risk of suicide-definition, drug safety concerns, and a necessary target for drug development: a consensus statement. J Clin Psychiaty.

[30] Bronisch T (1992). Does an attempted suicide actually have a cathartic effect?. Acta Psychiatr Scand.

[31] Davis AT, Schrueder C (1990). The prediction of suicide. Med J Aust.

